# A Natural Healer

**Published:** 1887-09

**Authors:** 


### A NATURAL HEALER.

A writer in the Boston Weekly Transcript, over the signature of Edward C. Towne, gives the following account of a celebrated healing medium located in that city, that of a Doctor. "whose sole means of effecting cures, in fully as large proportion as any known practice, and for remarkable cures in larger proportion than the best ordinary practice, is that of the electrical or magnetic energy which he communicates by the use of his hands, one of which has a much more intense effect than the other. He became a doctor against his will. A poor fellow whose hands were helpless, his fingers drawn together and unable to open, was sent to him, half in joke, to be cured ; and, yielding to his evidently honest importunity, Mr. B. tried, and was himself surprised by a cure wrought in a few moments. The noise which it made brought other cases so fast, that all other business had to be relinquished, and the practice of the first year yielded $3,000, in spite of the fact that very many cases were treated gratuitously. Now this Dr. B. refuses to treat cases which do not reveal themselves as open more or less to his power, and these, I believe, are about one-fourth of those brought to him. He would make much more to attempt all cases, but he knows when he cannot do any good, and refuses. Why the power will not work in these cases he does not know. In all cases where there is any chance he feels the power as soon as he sees the patient, and is usually guided to a knowledge of the trouble by what he feels, or by a purely mental impression. He sometimes gets this guidance before he sees the patient, and gets it so clearly as to feel the trouble himself for a moment. Thus, called to a man who had sprained his left ankle, he felt the sprain in his own left ankle as he went up the steps of the man's house, a momentary feeling only. In another case a woman came to him, ostensibly for a call Sunday afternoon, but intending to ask whether, as she feared, a tumor was threatening her life. Her courage to speak failed, and sitting across the room from the doctor, she made believe only a call. After a few moments, the doctor said to her, "This is not what you came for ; you needn't be alarmed about that swelling, it is not a tumor." This sort of experience, qualified of course, by a measure of unsatisfactory results, has gone on for more than ten years. A plain, homely, uneducated man, without advertising and without professional standing, has made a very large and beneficent success by simply using his hands for electrical and magnetic effects, which would pass for miraculous in an ignorant age. Experience with an immense variety of cases has taught him how to do his work, and yet he depends for each case upon the impressions which it makes, and cannot himself tell why these differ in what seem like cases. A distinct sensation is the sign by which he knows that he can treat a case, a sensation like that of a passing electrical charge. One hand gives apparently a mild negative current, and the other a powerful positive current, so that a sufferer from severe sciatica will be soothed by one hand, and made to howl with pain at the bare touch of the other, the current from which is exactly like a strong current from a battery. How many hopeless cases come to such a doctor, and what a margin of doubtful results there may be in such a practice, need not be pointed out, but with all this, the record of remarkable cures which it shows is none the less astonishing ; and such a practitioner, if he could have a large hospital and the aid of subordinates for a great part of the work (there being persons enough who have a good deal of the necessary power), would make as notable a mark as any of the great doctors of the time. One of his gratuitous cases not very long since, was that of a little girl treated without result for some weeks at the Massachusetts General Hospital, for paralysis of the nerves governing the valves of the veins. Treatment ten times effected a complete cure. I need not dwell on the work done by the doctor in question, as my only object here is to call attention to the extent to which impressions come to him, where the overwhelming presumption is that they are electrically transmitted, and that the whole of his remarkable power depends on electrical conditions."

